# Inhibition of Circ-Snrk ameliorates apoptosis and inflammation in acute kidney injury by regulating the MAPK pathway

**DOI:** 10.1080/0886022X.2022.2032746

**Published:** 2022-04-13

**Authors:** Fanhang Meng, Qiuyuan Chen, Shijie Gu, Ruiwen Cui, Qing Ma, Ronghua Cao, Ming Zhao

**Affiliations:** a Department of Organ Transplantation, Zhujiang Hospital of Southern Medical University, Guangzhou, China; b Department of Organ Transplantation, the Second Affiliated Hospital of Guangzhou University of Chinese Medicine, Guangzhou, China

**Keywords:** Acute kidney injury, circ-Snrk, MAPK pathway, ischemia and reperfusion, apoptosis, inflammation

## Abstract

**Background:**

Circular RNA (circRNA) is involved in the process of acute kidney injury (AKI), but only a few circRNAs have been reported. In the study, we investigated a new circRNA and its association with AKI.

**Methods:**

An AKI model was established in Sprague-Dawley rats, followed by serum creatinine and urea nitrogen tests measured by a biochemical analyzer. The pathological changes and apoptosis in the renal tissue were detected by Hematoxylin and Eosin, and TUNEL staining. Then, circRNA expression in AKI was determined by quantitative real-time-PCR (qRT-PCR). NRK-52E cells were induced with hypoxia/reoxygenation (H/R) as *in vitro* models and the circ-Snrk level was tested by qRT-PCR. The effects of circ-Snrk in H/R-induced NRK-52E cells were assessed by flow cytometry, western blot, and enzyme-linked immunosorbent assay. Finally, RNA sequencing and western blot analysis were used to validate the mRNA profile and pathways involved in circ-Snrk knockdown in H/R-induced NRK-52E.

**Results:**

A reliable AKI rat model and H/R cell model were established. qRT-PCR demonstrated that circ-Snrk level was upregulated in AKI left kidney tissue and NRK-52E cells with H/R treatment. Circ-Snrk knockdown inhibited apoptosis of NRK-52E cells and secretion of inflammatory factors (IL-6 and TNF-α). RNA sequencing showed that the mRNA profile changed after inhibition of circ-Snrk and differential expression of mRNA mainly enriched various signaling pathways, including MAPK signaling pathway. Furthermore, western blot indicated that circ-Snrk knockdown could inhibit the activation of p-JNK and p-38 transcription factors.

**Conclusions:**

Circ-Snrk is involved in AKI development and associated with the MAPK signaling pathway in AKI.

## Introduction

Acute kidney injury (AKI) reliably represents an abrupt deterioration of kidney function within 48 h among 2–7% of hospitalized patients [1,2] and is consistently caused by decreased renal blood flow and reperfusion injury [3]. Ischemia-reperfusion (I/R) injury of the kidney is a major cause of AKI, always associated with acute inflammation and progressive deterioration of renal function [4]. As a result, the AKI pathological process is often accompanied by local oxygen supply disorders, accumulation of metabolic waste, and renal tubular epithelial cell damage [5,6]. Specifically, renal tubular epithelial cell death ultimately results in irreversible renal function loss. Finally, these factors contribute to an abrupt decline in glomerular filtration rate (GFR), a sustained rise in serum creatinine (Cr) and blood urea nitrogen (BUN), and a reduction in urine output [5]. While the modulation of inflammatory response and inhibition of apoptosis are key factors in the preservation of renal function [7], their underlying driving mechanisms under the I/R are still unclear.

Circular RNAs (circRNAs) have been studied recently and are known to be associated with various diseases [8–10]. CircRNAs comprise a class of endogenous non-coding RNA (ncRNA) molecules that affect the miRNA functions through the competing endogenous RNA (ceRNA) network, suggesting that circRNAs can play a vital role in post-transcription [11]. The roles of circRNAs in cell apoptosis, oxidative stress, and inflammation indicate them as potential regulators in AKI [12–14]. However, circRNAs that are reportedly related to the pathogenesis of AKI need further investigation and validation. Additionally, circRNAs used for AKI biomarkers are limited. Furthermore, the specific mechanisms of circRNAs in the regulation of renal tubular epithelial cell function in the development of I/R-induced renal diseases require more studies.

In this study, we constructed AKI models *in vivo*, in which we compared the expression levels of five circRNAs based on the preexisting literature [15] and selected circ-Snrk for further observation. Furthermore, a reliable H/R NRK-52E cell model *in vitro* was established to observe the effect of circ-Snrk on apoptosis and inflammatory cytokines. We also performed whole-transcriptome sequencing to reveal a group of mRNAs differentially expressed after circ-Snrk knockdown. Furthermore, we investigated the role of circ-Snrk in the MAPK signaling pathway, which regulates the inflammatory cytokines and cell apoptosis involved in renal injury [16,17]. Our findings may offer novel insights into the role of circRNAs in AKI development and treatment, and also provided new clues for further study.

## Materials and methods

### Establishment of an AKI model

All experimental procedures were conducted in accordance with the Guide for the Care and Use of Laboratory Animals, with approval of the Ethics Committee of Guangzhou Forevergen Medical Laboratory Animal Center (Guangdong, China). Six Sprague-Dawley rats aged 6–8 weeks (200–250 g) were divided into two groups randomly using a random number table: The sham group and the AKI group. The IRI-induced AKI animal model was constructed by following the protocol of our previous study [18]. In brief, ischemia was induced by blocking the left renal vertebral arch and arteries for 45 min, and the removal of the right kidney. After 48 h of reperfusion, blood and left kidney tissue were collected for further study.

To investigate whether circ-Snrk had an effect on the IRI-induced AKI animal model, the lentivirus target for circ-Snrk (shRNA), as well as the negative control lentivirus (shRNA-NC), were purchased from Genepharma (Shanghai, China), Then another 15 Sprague-Dawley rats were used for this experiment and randomly divided into three groups (five rats per group): sham group, AKI + shRNA NC group, and AKI + shRNA group. For AKI + shRNA NC group and AKI + shRNA group, lentivirus was injected into tail vein with 1 × 10^9^TU following with previous study [19].

### Fluorescence in situ hybridization (FISH)

The FISH assay was used to ascertain the localization of circ-Snrk in the kidney. The probe of circ-Snrk was provided by RIBOBIO (Guangzhou, China). The FISH experiment was conducted following the protocol of fluorescent *in Situ* Hybridization Kit (RiboBio, Guangzhou, China). As a previous study showed that E-cadherin was expressed in the renal tubular epithelial cells [20], therefore we selected E-cadherin (#20874-1-AP; dilution: 1:100; Proteintech, Wuhan, China) to confirm the location of renal tubular epithelial cells in the kidney. At last, confocal laser-scanning microscopy (Zeiss, Germany) was used to investigate the expression of circ-Snrk and E-cadherin.

### Serum creatinine (Cr) and blood urea nitrogen (BUN) assay

Serum creatinine (Cr) and blood urea nitrogen (BUN) were detected using an automatic biochemical analyzer (BE-2000, Mindray) following the instructions of the kits (Roche).

### Hematoxylin-Eosin (HE) and TUNEL staining assay

The left kidney tissue samples were fixed with 10% formaldehyde for 24 h, then embedded in paraffin and sectioned (5 μm thickness). The sections were stained with a HE staining kit (Beyotime Biotechnology, Shanghai, China), then photographed with a Nikon Eclipse Ti-S microscope (Tokyo, Japan). The 5-μm-thick paraffin kidney sections were stained with a TUNEL staining kit (Thermo Fisher Scientific, Waltham, MA, USA), according to the manufacturer’s instructions, and observed using a Nikon Eclipse Ti-S microscope.

### Cell culture and treatment

NRK52E cells were purchased from Shanghai Cell Bank (Shanghai, China) and were cultured in Dulbecco’s modified Eagle’s medium supplemented with 5% fetal bovine serum (Gibco, Grand Island, NY, USA) and 50 units/mL penicillin/streptomycin (Gibco) at 37 °C in humidified air containing 5% CO_2_. The hypoxia-reoxygenation (H/R) cell model was constructed according to the protocol within a previous study [18].

### Cell viability assay

Cell viability of the NRK-52E cells was measured by using a Cell Counting Kit-8 (CCK-8) (Beyotime Biotechnology). Briefly, after treatment with H/R, the NRK-52E cells in the growth phase were seeded into 96-well plates at a density of 5000 cells/well. The cells were incubated at 37 °C, then 10 μL of CCK-8 reagent were added into each well and the cells were incubated for an additional three hours. The optical density at 450 nm was measured using a Microplate Reader (Thermo Fisher Scientific). The viability of the NRK-52E cells was measured at 24 h, 48 h, and 72 h.

### Cell apoptosis assay

Cell apoptosis was determined with Annexin V-APC/7-AAD Apoptosis Detection reagent (Dojindo Laboratories, Kumamoto, Japan). NRK-52E cells in the control group and the H/R group were collected, washed three times with binding buffer, and resuspended in binding buffer, followed by incubation with Annexin V-APC and 7-AAD for 15 min at room temperature in the dark. Apoptotic cells were quantified by flow cytometry (Dako, Ely, UK). Annexin V+/7-AAD − cells were considered to undergo the early stage of apoptosis and cells with Annexin V+/7-AAD + were considered to undergo the end stage of apoptosis.

### Isolation of RNA and quantitative RT-PCR (qRT-PCR)

RNA was extracted using TRIzol reagent (Thermo Fisher Scientific) or Hipure Liquid RNA Mini Kit (Magen, St. Louis, MO, USA) for plasma according to the manufacturer's instructions. The integrity and concentration of the RNA samples were determined using agarose gel electrophoresis and the NanoDrop ND-1000 (NanoDrop Technologies, DE, USA). The RNA was reverse-transcribed into cDNA using the TaqMan MicroRNA reverse transcription kit (Ambion, CA, USA). The expression of circRNA was measured by qRT-PCR using the GoTaq qPCR Master Mix (Promega, Madison, WI, USA). Data were analyzed based on the ΔCT or 2^−ΔΔCT^method, and GAPDH was used as an internal control. The primers of this study are included in Table 1.

**Table 1.** Primer sequence.

Gene name	Primer sequence (5’–3’)
Rno-circ-Eya3-diver-F	CACATCCTCTCGGTTCCTGT
Rno-circ-Eya3-diver-R	CGGTCTTCACAAACTGTCGA
Rno-circ-Memo1-diver-F	ACGGAGAGCTATGGAAGACAG
Rno-circ-Memo1-diver-R	CTGAGGTCCTTTCCATGGCT
Rno-circ-Snrk-diver-F	CTTGGCGATGAGTACGATGC
Rno-circ-Snrk-diver-R	TGCTGGTTCAATGAAGGGGA
Rno-circ-Snrk-conver-F	AGCCTGGAAAGAAGCTCACT
Rno-circ-Snrk-conver-R	ATGGATACCTTGCAAAACTGC
Rno-circ-Nmt1-diver-F	GTTTCTCTTGTGGTGAAGTGGT
Rno-circ-Nmt1-diver-R	CCAAGGCATCCCAAGTGAAG
Rno-circ-Co14a1-diver-F	ATGAAGGGGCAGAAAGGAGA
Rno-circ-Co14a1-diver-R	GCCCGACATCACCTTTATCAC
GAPDH-diver-F	GAGTCAACGGATTTGGTCGT
GAPDH-diver-R	GAGTCAACGGATTTGGTCGT
GAPDH-conver-F	TCCTCACAGTTGCCATGTAGACCC
GAPDH-conver-R	TGCGGGCTCAATTTATAGAAACCGGG

### Electrophoresis and sequencing of qRT-PCR products

The divergent and convergent primers designed for the circRNAs, as well as exon region amplification, were shown in Table 1. Next, the PCR products were proved by 1.5% agarose gel electrophoresis. Subsequently, the divergent amplified product was subjected to Sanger sequencing for circ-Snrk conjunction structure identification.

### Inflammatory factors levels assay

The inflammatory factor levels in the cell supernatant were measured by enzyme-linked immunosorbent assay (ELISA) kits (Cuabio, Wuhan, China), according to the manufacturer’s instructions.

### Total protein extraction and Western blot analysis

Total protein from the NRK-52E cells was extracted with radioimmunoprecipitation assay (RIPA) buffer (Thermo Fisher Scientific). Then, the proteins were separated *via* 10% sodium dodecyl sulfate-polyacrylamide gel electrophoresis and transferred to polyvinylidene difluoride (PVDF) membranes. The membranes were blocked with 5% nonfat milk for 2 h at room temperature. Next, the membranes were incubated with primary antibodies at 4 °C overnight, followed by incubation with secondary antibodies. The protein expression levels were measured by an enhanced chemiluminescence detection kit (Bio‐Rad, Hercules, CA, USA) and the bands were observed using a gel imaging system (Odyssey, LI‐COR Biosciences). The primary antibodies used were as follows: anti-cleaved caspase3 (#9662; dilution: 1:1000; Cell Signaling Technology, Danvers, MA, USA), anti-Bcl-2-associated X protein (Bax, #50599-2; dilution: 1:1000; Proteintech), p-JNK (#4668; dilution: 1:1000; Cell Signaling Technology), JNK (#9258; dilution: 1:1000; Cell Signaling Technology), p-P38 (#4511; dilution: 1:1000; Cell Signaling Technology), P38 (#8690; dilution: 1:1000; Cell Signaling Technology), p-ERK (#AF-1018; dilution:1:2000; R&D, Minnesota, MN, USA), ERK (#AF-1576-SP; dilution:1:1000; R&D), and anti-GAPDH (#60004-1; dilution: 1:8000; Proteintech).

### Circ-Snrk siRNAs transfection

For knockdown of circ-Snrk in NRK52E cells, three siRNA (siRNA-1, si-RNA-2, siRNA-3) target sequences and one control sequence (siRNA-NC) were purchased from Genepharma (Shanghai, China). Lipofectamine 2000 (Invitrogen, Carlsbad, CA) was used for cell transfection, according to the manufacturer’s instructions. After transfection for 48 h, the cells were collected and examined by qRT-PCR. The most efficient circ-Snrk siRNA was used for further investigation. After transfection for 48 h, the cells were treated with H/R and collected for further study. The siRNA sequences are listed in Table 2.

**Table 2.** siRNA sequence in the study.

siRNA name	Sense (5'-3')	Antisense (5'-3')
siRNA-1	GCAGUAGGAGAAACAGCAA	UUGCUGUUUCUCCUACUGC
siRNA-2	UACGAUGCCCCUGCAGUAGGA	UCCUACUGCAGGGGCAUCGUA
siRNA-3	GCCCCUGCAGUAGGAGAAA	UUUCUCCUACUGCAGGGGC
siRNA-NC	AGGAGGGCAAACACGAAAA	UUUUCGUGUUUGCCCUCCU

### RNA-Seq

The NRK-52E cells underwent transfection with siRNA-1 or siRNA-NC and then were treated with H/R. RNA was isolated and enriched by magnetic bead Oligo-dT (Dynal Biotech, Oslo, Norway). After RNA fragmentation, [the RNA samples underwent] random hexamer primed reverse transcription and adapter ligation. The generated libraries were examined by Agilent Technologies 2100 Bioanalyzer (Agilent Technologies, Germany) and subjected to sequencing on an Illumina HiSeq2000 instrument (Illumina, San Diego, CA). Next, the clean data was obtained after raw data filtering, and aligned to the hg19 human reference genome using Short Oligonucleotide Analysis Package alignment software. Read counts were analyzed, and differentially expressed mRNAs were screened based on *P*-value (<0.05) and absolute value of fold change (|FC|) >1.5. Hierarchical cluster analysis was created by the heatmap package in R software and the scatter plot was drawn using R software.

### Kyoto encyclopedia of genes and genomes (KEGG) enrichment analysis

We also visualized differentially expressed genes on the Database for Annotation, Visualization and Integrated Discovery (http://david.abcc.ncifcrf.gov/) pathway map to explore the biological pathways. KEGG terms were enriched with the cutoff criteria of *p* < 0.05.

### Statistical analysis

Data statistical analysis was performed using SPSS software (version 17.0 software: SPSS, Chicago, IL, USA). Values are presented as the mean ± standard deviation (SD). The 2-sample comparison was performed using the Mann-Whitney *U* test, and the one-way ANOVA with turkey post-hoc test was used for comparison between the various samples. *p* < 0.05 was established as a statistically significant difference.

## Results

### Establishment of the rat AKI model by I/R treatment

**Figure 1.**
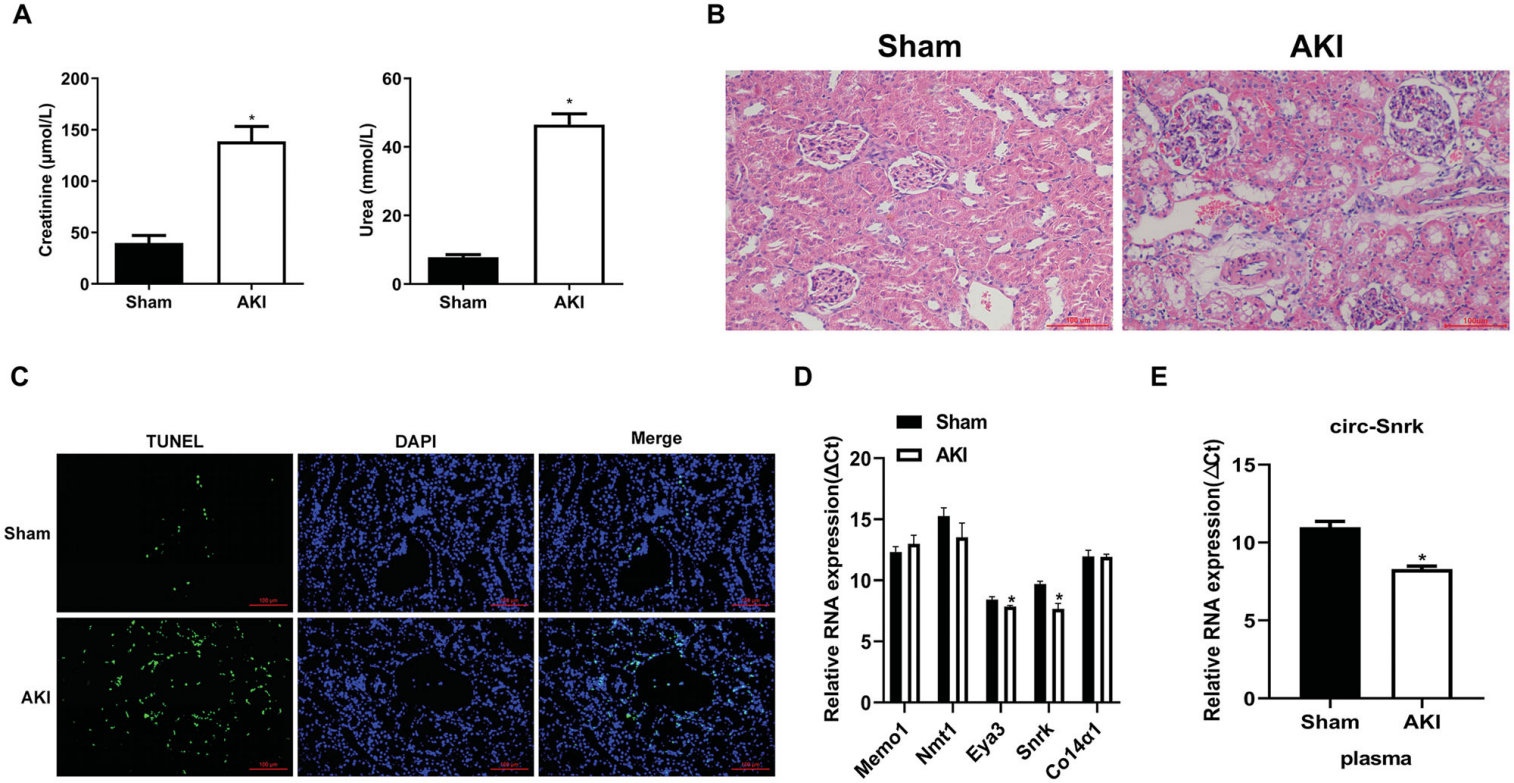
Preliminary validation of the five selected circular RNAs in I/R-induced acute kidney injury. (A) The levels of serum creatinine (Cr) and blood urea nitrogen (BUN) were significantly elevated in acute kidney injury (AKI) within the left kidney tissues, induced by ischemia and reperfusion. Sham group (*n* = 3), AKI group (*n* = 3). (B) A hematoxylin and eosin staining assay of the left kidney tissues was performed to estimate the kidney injury. Scale bar = 50 μm. (C) TUNEL staining of left kidney tissues to estimate the apoptosis. Green, TUNEL; blue, DAPI; scale bar = 200 μm. (D) The expression levels of circ-Memo1, circ-Nmt1, circ-Eya3, circ-Snrk, and circ-Co14α1 were validated by qRT-PCR in left kidney tissues. (E) The expression levels of circ-Snrk in plasma. The ΔCT values were determined by subtracting the CT values of GAPDH from the CT values of circRNAs. A larger ΔCT value indicates lower expression. Data were presented as mean ± SD. Each experiment was independently repeated at least three times. *P < 0.05 and **P < 0.01 vs. sham group. AKI, acute kidney injury.

To explore the role of circRNAs in AKI, the rat AKI model induced by IRI was constructed. As shown in Figure 1(A), the serum creatinine and BUN significantly and rapidly increased in the AKI group compared with the sham group. Then, the HE stains and TUNEL assay were performed to assess the kidney injury severity. HE results showed that the rats in the AKI group exhibited obvious renal tubular dilation, cast formation, cell necrosis, and inflammatory infiltration (Figure 1(B)). The TUNEL staining also indicated that TUNEL-positive cells had significantly increased in the left kidney tissues of the AKI group (Figure 1(C)). These results demonstrated that we have successfully constructed an AKI model that can be used for further validation of the potential targeted circRNAs’ expression. Consistent with earlier reports [15], circ-Eya3 and circ-Snrk were increased in the IRI induced AKI group (decrease in ΔCT), compared to the control group (Figure 1(D)), and circ-Snrk increased much more than circ-Eya3. However, three additional circRNAs, circ-Memo1, circ-Nmt1, and circ-Co14α1, showed no variation in expression between the two groups. Therefore, circ-Snrk was selected for further validation in plasma, and increased circ-Snrk expression was found in the AKI group (Figure 1(E)).

### Circ-Snrk is upregulated in H/R NRK-52E cells

**Figure 2.**
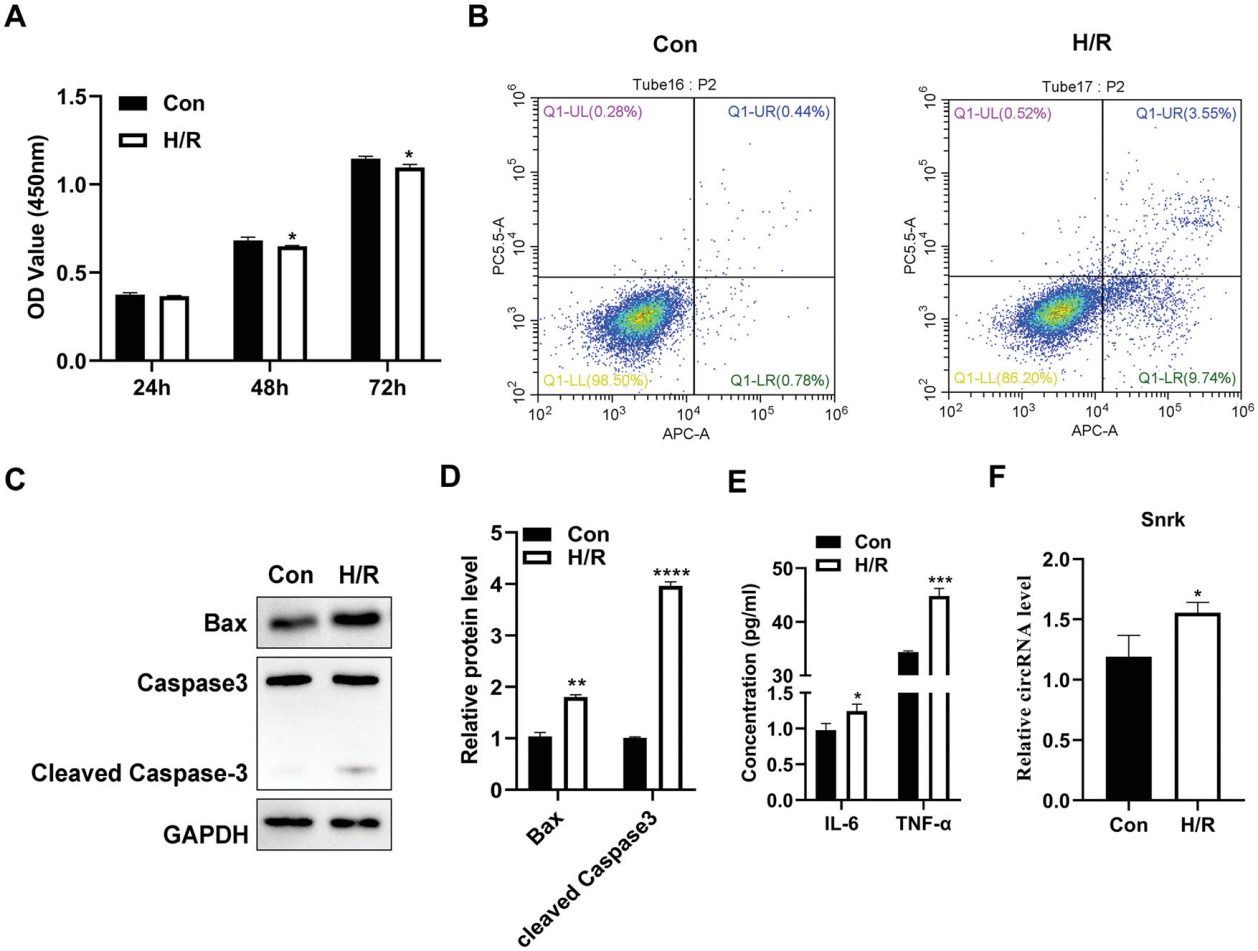
Circ-Snrk is upregulated in H/R-treated NRK-52E cells. (A) A CCK-8 assay was performed to assess the cell viability in the H/R-treated NRK-52E cells and the control group. (B) Flow cytometry was performed to measure the apoptosis ratio in the H/R-treated NRK-52E cells and the control group. (C) Western blot analysis was performed to measure the levels of apoptosis-promoting proteins BAX and cleaved caspase 3 in the H/R-treated NRK-52E cells and the control group. (D) The relative protein expression analysis of (C). (E) An ELISA assay was used to determine the levels of the pro-inflammatory cytokines, IL-6 and TNF-α, within the supernatant of the H/R-treated NRK-52E cells and the control group. (F) A qRT-PCR assay was used to measure the relative expression levels of circ-Snrk in H/R-treated cells and the control group. *P < 0.05, **P < 0.01, ***P < 0.001, ****P < 0.0001 vs. control group.

To further investigate whether circ-Snrk is involved in IRI-induced AKI, we performed H/R treatment on NRK-52E cells (reoxygenation for 6 h after hypoxia for 24 h) for the establishment of the H/R-induced AKI cell model. Cell viability was determined using the CCK-8 assay and presented results that compared with the control group (Con); the viability of NRK-52E cells was inhibited in the H/R group (Figure 2(A)). The flow cytometry assay also demonstrated an increased cell apoptosis ratio after H/R treatment (Figure 2(B)). Consistently, H/R treatment significantly upregulated the apoptosis-promoting gene *BAX* and cleaved caspase3 protein levels (Figure 2(C,D)). In addition, the concentration of pro-inflammatory cytokines IL-6 and TNF-α also increased in the H/R group compared with the control group (Figure 2(E)). All the results *in vitro* were consistent with the results obtained from the renal I/R model *in vivo*, demonstrating that the H/R cell model was established successfully. Moreover, the relative expression level of circ-Snrk in the H/R group was significantly higher than that of the control group (Figure 2(F)), which was also consistent with the results *in vivo*.

### Circ-Snrk knockdown reduced the secretion of inflammatory factors and inhibited apoptosis in H/R-Treated NRK-52E cells

**Figure 3.**
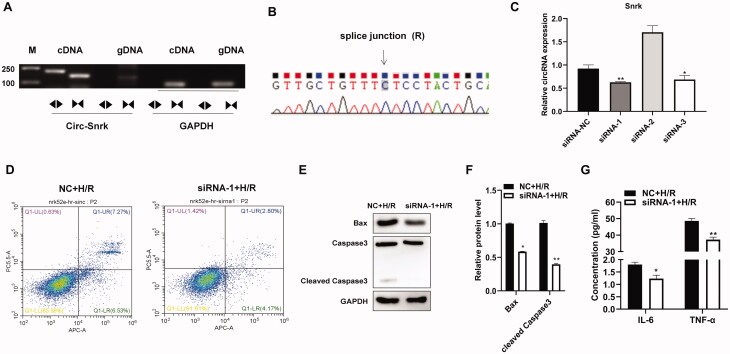
Inhibition of circ-Snrk inhibits apoptosis and secretion of inflammatory factors induced by H/R in NRK-52E cells. (A) Electrophoresis of RT-PCR products. Divergent primers detect circular RNAs in cDNA but not genomic DNA (gDNA). GAPDH was used as an internal reference. Lane M is the marker; lanes are PCR products. (B) Validation of circ-Snrk splicing junction site through Sanger sequencing. (C) The qRT-PCR analysis of three siRNAs’ knockdown efficiency of circ-Snrk in NRK-52E cells. (D) Apoptotic cells were measured by flow cytometry using an Annexin V-APC/7-AAD apoptosis kit. (E) Western blotting showed BAX and cleaved caspase3 protein levels reduced in the siRNA-1 + H/R group compared to the NC + H/R group. (G) An ELISA assay showed that knockdown of circ-Snrk could reduce the secretion of inflammatory factors IL-6 and TNF-α in the siRNA-1 + H/R group compared to the NC + H/R group. Data are given as mean ± SD (n = 3; t-test). *P < 0.05, **P < 0.01.

To further investigate the function of circ-Snrk, we first verified the circuitry of circ-Snrk by RT-PCR and Sanger sequencing. The RT-PCR products of convergent and divergent primers were validated by 1% agarose gel electrophoresis, with results showing that only circ-Snrk divergent primers could amplify the products from cDNA (Figure 3(A)). In addition, the Sanger sequencing also verified the junction site of the circ-Snrk (Figure 3(B)). The above results verified the structure of circRNA. Then, we knocked down the expression of circ-Snrk in the NRK-52E cells *via* transfection with siRNAs. The knockdown efficiency was confirmed by qRT-PCR after cell transfection for 48 h. As shown in Figure 3(C), siRNA-1 and siRNA-3 decreased circ-Snrk expression in the NRK-52E cells. The siRNA-1 was more efficient than siRNA-3, so siRNA-1 was used for further study. In the siRNA-1 + H/R group, circ-Snrk knockdown could significantly inhibit apoptosis and reduce the protein expressions of the pro-apoptotic proteins, BAX and cleaved caspase 3, compared with the NC + H/R group (Figure 3(D–F)). To further explore the inflammatory response in NRK-52E cells caused by H/R, the secretions of inflammatory factors IL-6 and TNF-α in the supernatant were subsequently collected for detection by ELISA. The levels corresponding to these factors significantly decreased in the siRNA-1 + H/R group compared to the NC + H/R group (Figure 3(G)). These results suggest that down-regulation of circ-Snrk expression could inhibit apoptosis and reduce inflammatory factors’ secretions in H/R-induced NRK-52E cells.

### Alternation of the mRNA expression profile after Circ-Snrk knockdown and H/R treatment in NRK-52E cells

**Figure 4.**
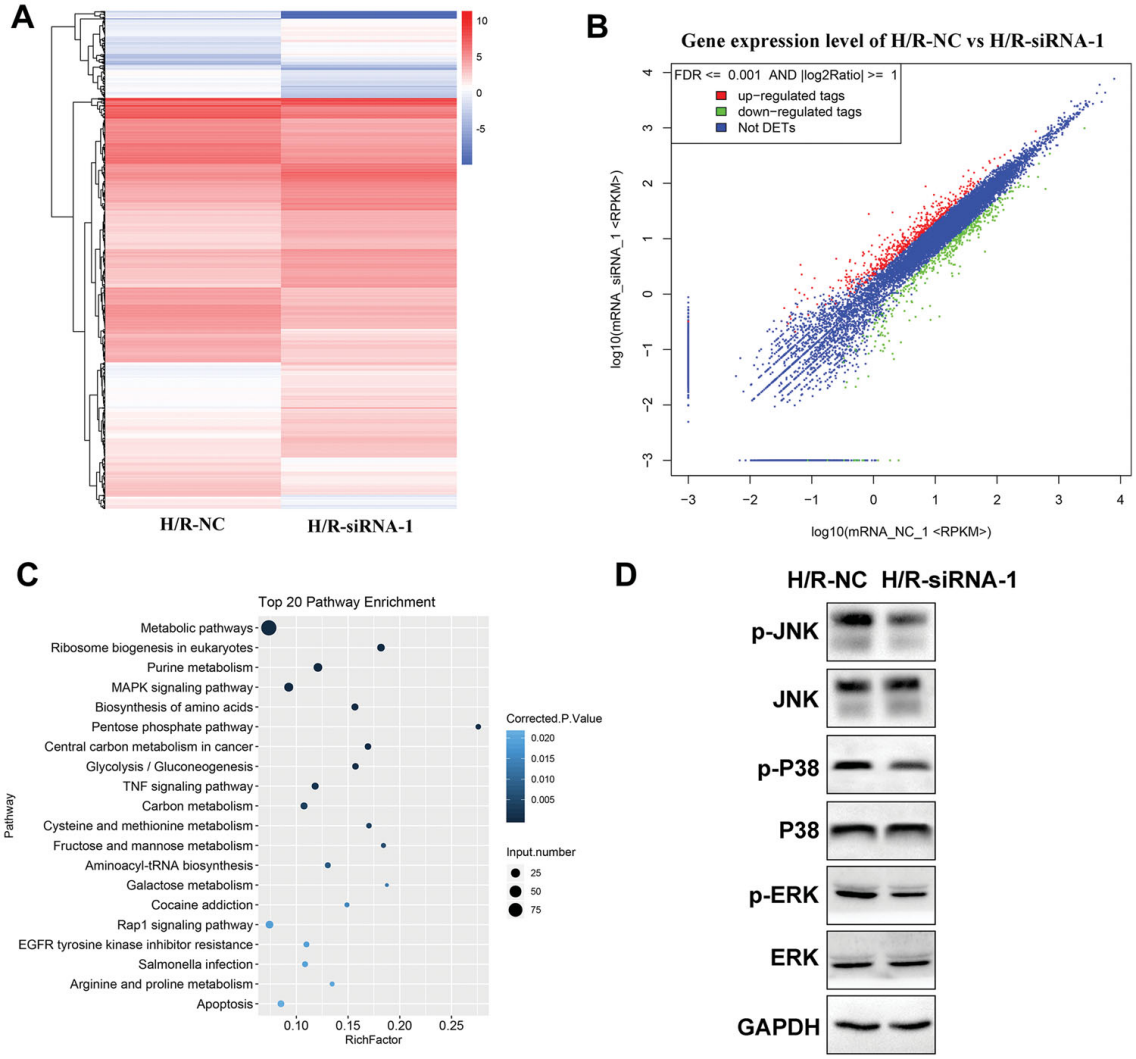
Circ-Snrk knockdown inhibits the H/R-activated MAPK pathway. (A) Hierarchical cluster heat map and (B) scatter plots are used to evaluate the difference in the expression of mRNAs between the NC + H/R and siRNA-1 + H/R groups. The green plots indicate down-regulated genes; the red plots indicate upregulated genes. (C) Top 20 enriched pathways of differently expressed mRNAs by KEGG analysis. D. The effects of circ-Snrk knockdown on MAPK pathway-related key protein levels were detected in cells of the NC + H/R and siRNA-1 + H/R groups via western blot.

To further elucidate the molecular mechanisms of circ-Snrk mediation on H/R-treated NRK-52E cells, we tested the mRNA expression profile of H/R-treated NRK-52E cells after circ-Snrk knockdown through RNA-seq. As illustrated in Figure 4(A), the hierarchical clustering analysis of differentially expressed mRNAs suggested that a total of 202 mRNAs were upregulated while 75 were down-regulated (|fold change| >1.5, *p* < 0.05) in the siRNA-1 + H/R group, as compared to the NC + H/R group. Additionally, the differential expression of the mRNAs was evident in the scatter plot (Figure 4(B)). Further examination of the differentially expressed mRNAs took place through KEGG analysis. The top 20 KEGG pathways were summarized in Figure 4(C). Significantly enriched KEGG pathways included the MAPK signaling pathway, TNF signaling pathway, Rap1 signaling pathway, EGFR tyrosine kinase inhibitor resistance, and apoptosis. Based on the results, we further investigated the MAPK pathway-related proteins in the cell model. Western blot analysis revealed that phosphorylated p38 (p-p38), phosphorylated JNK (p-JNK), and phosphorylated ERK (p-ERK) expression levels were decreased in the siRNA-1 + H/R group, as compared to the NC + H/R group (Figure 4(D)).

### Circ-Snrk knockdown decreased the kidney injury severity

**Figure 5.**
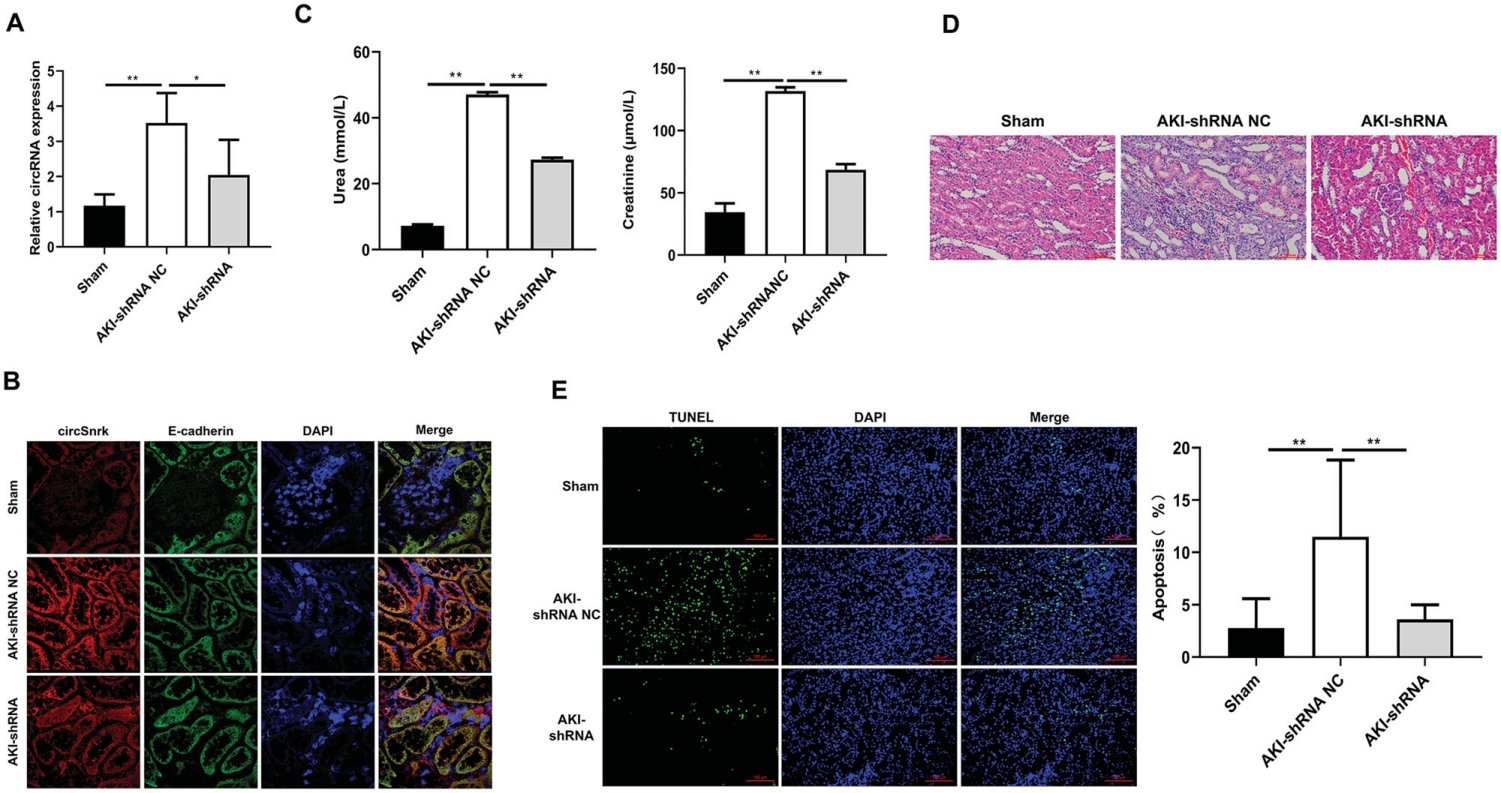
Circ-Snrk Knockdown decreased the left kidney injury severity. (A) qRT-PCR was used to evaluate the relative expression of circ-Snrk expression in the left kidney. Sham group (*n* = 5), AKI + shRNA NC group (*n* = 5), and AKI + shRNA group (*n* = 5). (B) The location of circ-Snrk in the kidney was detected using the FISH experiment, and E-cadherin was used to indicate the location of renal tubular epithelial cells. (C) The levels of serum creatinine (Cr) and blood urea nitrogen (BUN) in the left kidney were measured in the Sham group, AKI + shRNA NC group, and AKI + shRNA group. (D) hematoxylin and eosin staining assay of the left kidney tissues was performed to estimate the kidney injury. Scale bar = 50 μm. (E) TUNEL staining of left kidney tissues to estimate the apoptosis. Green, TUNEL; blue, DAPI; scale bar = 200 μm.

Firstly, the relative expression of circ-Snrk in left kidney tissues was detected using qRT-PCR. Results showed that circ-Snrk expression level was promoted in AKI + shRNA NC group relative to those in sham group; and circ-Snrk expression was inhibited in AKI + shRNA group compared to the AKI + shRNA NC group (Figure 5(A)). These results confirmed that lentivirus containing circ-Snrk shRNA could effectively decrease the circ-Snrk expression in the left kidney of rats. In addition, we also confirmed the circ-Snrk was localized in the renal tubular epithelial cells by FISH (Figure 5(B)). Knockdown of circ-Snrk inhibited the increase by AKI-induced serum creatinine and BUN (Figure 5(C)). Same with the results in Figure1B,C, compared to the sham group, AKI + shRNA NC group showed obvious renal tubular dilation, cast formation, cell necrosis, and inflammatory infiltration (Figure 5(D)), moreover, the TUNEL-positive cells had significantly increased (Figure 5(E)). However, inhibition of circ-Snrk decreased the inflammatory infiltration and the TUNEL-positive cells compared to AKI + shRNA NC group (Figure 5(D–E)).

## Discussion

In this study, we found that serum creatinine and BUN significantly increased in the I/R-induced rat AKI model. Additionally, the rat model exhibited an increase in left kidney pathological injury and apoptosis, as compared with the sham group. I/R treatment of the NRK-52E cells decreased cell proliferation, increased cell apoptosis, and increased the secretions of IL-6 and TNF-α. Circ-Snrk was verifiably upregulated with I/R treatment compared to the untreated group. Inhibition of circ-Snrk decreased apoptosis, as well as the secretions of IL-6 and TNF-α. Furthermore, circ-Snrk knockdown altered the mRNA profile, and the differential expression of mRNAs mainly enriched some pathways, including the MAPK signaling pathway, which were further verified was inhibited after circ-Snrk knockdown under I/R treatment.

I/R injury, which inevitably occurs in renal tissue during surgery involving renal or aortic vascular occlusion, is the leading cause of perioperative AKI and is consistently associated with extremely high mortality and morbidity [21]. AKI is characterized by an increased serum Cr level, decreased GFR, and reduction of urine output due to a serious inflammatory response accompanied by epithelial cell apoptosis. The crosstalk associated with oxidative stress, inflammation, and epithelial cell apoptosis has been shown to play a vital role in AKI [22]. In this study, we demonstrated a rat AKI model with increased serum creatinine and BUN, and the left kidney was observed to exhibit severe pathological injury and apoptosis. High levels of creatinine and urea in the serum reflected a decrease in the GFR, suggesting loss in renal function. Tubular cell apoptosis is a primary and major contributor to the pathophysiology of renal I/R and determined the outcome of the renal damage. Additionally, the H/R-induced NRK-52E cell model was also constructed, resulting in decreased proliferation, increased apoptosis, and increased secretion of inflammatory factors IL-6 and TNF-α. All these results indicate that we established reliable models *in vivo* and *in vitro*, in which we could investigate the effects of circRNA expression.

Studies have indicated that circRNAs are involved in the progression of AKI [23,24]. A recent study showed that ciRs-126 was highly suppressed in AKI patients and could act as an independent predictor of 28-day survival [23]. Most circRNAs act as miRNA/lncRNA sponges to regulate mRNA expression. A previous study found serval dysregulated circRNAs between the sham and AKI rat groups by RNA-sequencing. Among the top 20 dysregulated circRNAs in this study, we validated five circRNAs (circ-Memo1, circ-Nmt1, circ-Eya3, circ-Snrk, and circ-Co14α1) by qRT-PCR. Among the five validated circRNAs, only circ-Eya3 and circ-Snrk expression levels were increased in our research, with circ-Snrk being the most markedly elevated [15]. Moreover, the host gene of circ-Snrk, *SNRK*, was observed to participate in AKI development [25]. However, the underlying molecular mechanisms of the effects of circ-Snrk in the AKI process remain unknown. Therefore, we selected circ-Snrk for further research. Our study identified that circ-Snrk expression increased in both the *in vivo* and *in vitro* models, a result that was consistent with the previous study [15].

Studies have shown that abnormal activation of NF-κB and MAPKs may promote the transcription of pro-inflammatory factors, including TNF-α, IL-1β, and IL-6 [26]. In addition, TNF-α and IL-6 also activate MAPKs, further promoting inflammatory reactions and apoptosis [27]. Therefore, inhibition of the activation of the MAPK signaling pathway could decrease the secretion of pro-inflammatory factors, including TNF-α, IL-1β, and IL-6, and the decreased secretion of pro-inflammatory factors could subsequently inhibit the activation of the MAPK signaling pathway. In our study, we found that circ-Snrk knockdown could reverse the cell apoptosis, pro-inflammatory cytokine (IL-6 and TNF-α) secretion, and the activation of the MAPK signaling pathway in the H/R-treated renal tubular epithelial cells NRK-52E, which indicated that circ-Snrk may be a novel target to treat AKI. Moreover, knockdown of circ-Snrk could reverse the effect of AKI-induced increase of serum creatinine, serum urea nitrogen, inflammatory infiltration, and apoptosis *in vivo*.

Our study has several limitations. The role of circ-Snrk was only tested in a cell model, with the only expression level validation in an *in vivo* model. The data from patients and *in vivo* conditions is still lacking. In addition, the potential mechanism of circ-Snrk regulation corresponding to the MAPK signaling pathway requires further investigation. Moreover, the effect of Circ-Snrk on inflammatory cells including macrophages, T lymphocytes and neutrophils infiltration also needs further study as the circ-Snrk expression to affect the expression of the pro-inflammatory factor.

## Conclusion

In this study, we established an I/R-induced rat AKI model *in vivo* and a H/R-induced cell model *in vitro* and identified up-regulation of circ-Snrk in both models. We demonstrated that circ-Snrk knockdown could inhibit NRK-52E cells’ apoptosis and inflammatory response in the H/R-induced cell model. Furthermore, the MAPK signaling pathway was verified to be involved in circ-Snrk regulation of the AKI process. These findings offer new clues for further study.

## Data Availability

The data that support the findings of this study are available from the corresponding author by reasonable request.
